# Pharmacokinetic/Pharmacodynamic Modeling and Application in Antibacterial and Antifungal Pharmacotherapy: A Narrative Review

**DOI:** 10.3390/antibiotics11080986

**Published:** 2022-07-22

**Authors:** Laiz Campos Pereira, Marcelo Aguiar de Fátima, Valdeene Vieira Santos, Carolina Magalhães Brandão, Izabel Almeida Alves, Francine Johansson Azeredo

**Affiliations:** 1 Laboratory of Pharmacokinetics and Pharmacometrics, Faculty of Pharmacy, Federal University of Bahia (UFBA), Rua Barão de Jeremoabo, 147, Salvador 40170-115, BA, Brazil; laiz.campos08@gmail.com (L.C.P.); marcelodefatima@hotmail.com (M.A.d.F.); valdeenevieira19@gmail.com (V.V.S.); carolinambrandao@gmail.com (C.M.B.); izabel.alves@ufba.br (I.A.A.); 2 Pharmacy Graduate Program, Federal University of Bahia, Rua Barão de Jeremoabo, 147, Salvador 40170-115, BA, Brazil; 3 Center for Pharmacometrics & Systems Pharmacology, Department of Pharmaceutics, College of Pharmacy, Orlando, FL 328827, USA

**Keywords:** PK/PD modeling, antibacterial, antifungal, pharmacotherapeutic treatment

## Abstract

Pharmacokinetics and pharmacodynamics are areas in pharmacology related to different themes in the pharmaceutical sciences, including therapeutic drug monitoring and different stages of drug development. Although the knowledge of these disciplines is essential, they have historically been treated separately. While pharmacokinetics was limited to describing the time course of plasma concentrations after administering a drug-dose, pharmacodynamics describes the intensity of the response to these concentrations. In the last decades, the concept of pharmacokinetic/pharmacodynamic modeling (PK/PD) emerged, which seeks to establish mathematical models to describe the complete time course of the dose-response relationship. The integration of these two fields has had applications in optimizing dose regimens in treating antibacterial and antifungals. The anti-infective PK/PD models predict the relationship between different dosing regimens and their pharmacological activity. The reviewed studies show that PK/PD modeling is an essential and efficient tool for a better understanding of the pharmacological activity of antibacterial and antifungal agents.

## 1. Introduction

The discovery and production of antimicrobials in the early twentieth century is one of the most outstanding achievements of public health since infectious diseases were considered one of the leading causes of the high mortality rates [1]. Bacterial infections have a higher prevalence in intensive care units (ICU) and are one of the leading causes of mortality in these units, both in the adult and pediatric populations [2,3]. Furthermore, antibiotic resistance has been considered an expanding global crisis. Resistant bacteria have several mechanisms that prevent the proper action of the antibacterial [4].

Fungal infections have increased considerably in recent years and are associated with high mortality and morbidity rates, mainly in immunocompromised patients. Limitations in antifungal treatment, such as fungal resistance, unwanted side effects due to drug toxicity, and reduced spectrum of action, contribute to the aggravation of this type of infection [5].

Therefore, drug development with different mechanisms of action against bacteria and fungi is of paramount importance. The administration in the correct dose and dosage according to the specificity of each population is also a necessity, mainly due to the increased resistance of these microorganisms to most existing antimicrobials [6,7]. Another critical tool for improving pharmacological treatment is therapeutic drug monitoring (TDM). TDM refers to the individualization of a patient’s dosage regimen to attain drug plasma levels within the therapeutic range. TDM is also recommended for drugs with marked interindividual pharmacokinetic variability, no matter their therapeutic index [8]. 

Pharmacokinetics and pharmacodynamics knowledge is applied in the research and development of drugs and TDM. In fact, many candidate molecules fail in drug development because they have undesirable pharmacokinetic characteristics, such as very short or very high half-life (t_1/2_), poor absorption, or extensive first-pass metabolism. This leads, for example, to rationality in promoting changes in the compound’s chemical structure to improve these kinetic parameters [9]. Furthermore, pharmacotherapeutic monitoring assumes a direct relationship between administered dose, plasma concentrations, and pharmacological effects (the expected response). It only happens for drugs with a better relationship between plasma concentration and response, favoring the pharmacodynamic interpretation in TDM [10].

The areas of pharmacokinetics and pharmacodynamics had been treated in an isolated way: pharmacokinetics was limited to describing the time course of drug concentrations in different body fluids after administration of a dose, while pharmacodynamics had a description of the intensity of the response drug according to their concentrations at the site of action. However, pharmacokinetic studies only make sense if there is prior knowledge of an association between drug concentrations and their effects (therapeutic or adverse). In contrast, pharmacodynamic studies do not consider the time course of the concentration-effect relationships by assuming that drug concentrations at the site of action remain constant [11].

Thus, the concept of pharmacokinetic/pharmacodynamic modeling (PK/PD) emerged by applying mathematical models to describe the relationship between dose and drug response measured over time. The PK/PD models have a pharmacokinetic and a pharmacodynamic component; that is, they combine models of the first (e.g., mono and bicompartmental) with models of the second (e.g., model with fixed, linear, maximum effect, maximum sigmoidal effect) and, also, its data and correlations can be treated separately or simultaneously [11,12].

PK/PD models can potentially benefit all phases of drug development, including preclinical and clinical phases I, II, III, and IV. During phase I clinical trials, PK/PD models can support optimal dose and dosage regimen definition, in addition to potential biomarkers; accelerate the selection of candidate compounds; and assist in the prediction of oral bioavailability, the potency of the new drug (EC_50_), and its intrinsic activity, saving time and money in the development of the new drug. The developed model can then be improved and integrated with Phase I clinical studies, in which drug-drug interactions and the safety profile (toxicity) are evaluated. During phases II and III, PK/PD modeling is used to simulate clinical outcomes; assess the impact of covariables on the model (such as subpopulations of patients and comorbidities), and mainly confirm the dose-response relationships in the studied populations [13] as well as it can contribute to the technological development of new drug delivery systems [14]. 

Antimicrobials are associated with both the success (cure of infection) and the failure of the therapy (adverse effects, antimicrobial resistance, inability to resolve the infection), and the choice of appropriate dosage and dose translates into a rational decision in the clinical practice. The integration between pharmacokinetics and pharmacodynamics has been addressed in several studies aiming to use PK/PD models in rationalizing and individualizing dose regimes with antimicrobials. When using PK/PD modeling in pharmacotherapy, the results allow better decisions regarding the best antimicrobial for a patient and estimate the probability of success with the chosen posology and dose, contributing to the dissemination of antimicrobial resistance [15,16].

The use of PK/PD modeling in the developing antibacterials is considered a suitable approach that enables the optimization of study designs, reduced costs, and a shorter duration of clinical studies. In addition, the results obtained from studies with PK/PD modeling serve as support in the clinical area, in decision-making in situations with patients from specific populations who present higher variability in pharmacokinetics or due to the complexity in the treatment of infections caused by pathogens considered highly resistant [17,18].

Considering the positive impacts of the application of PK/PD models and simulations in drug development and precision medicine, this work aimed to review the main topics related to this field of pharmaceutical knowledge, the main approaches, and the future of PK/PD modeling concerning antimicrobial drug therapy. The concept and mathematical rationale of PK/PD models using the time-kill curves approach were reviewed for anti-infectives.

## 2. Basic Aspects of PK/PD Modeling

The rationale behind PK/PD modeling is to promote the union between the pharmacokinetics and pharmacodynamics of a drug so that it is possible to establish a dose-concentration-response relationship and predict the complete time course of a dose. For this, kinetic and pharmacodynamic models are used [19].

In PK/PD modeling, the kinetic model features components describing the time course of drug concentration obtained in body fluid samples after administration. The compartmental-type kinetic models are preferably used, as they provide a continuous profile of the concentration-time relationship. Preference should be given to modeling free unbound concentrations as they are responsible for the pharmacological effect, especially if there is suspicion of any non-linearity in the plasma or binding to the tissue that may interfere in the characterization of the dose-concentration-effect relationship [11].

Among the kinetic models, is the monocompartmental model, in which the human body is seen as a single central compartment, and the drug is presumed to be distributed homogeneously and quickly to all tissues. It is considered the simplest kinetic model, as it does not assume that the organism is composed of several tissues and organs that must be treated as distinct compartments [20]. Given this, multicompartmental models allow incorporating two or more compartments in addition to the central (plasma). This model is based on the ability of the drug to distribute quickly or slowly in some tissues; thus, the kinetic description is more appropriate when including one or more compartments called peripheral. However, bicompartmental models are more used since many drugs follow this kinetics [21].

The PK/PD modeling uses pharmacodynamic models, which relate the concentration to the response. In simpler models, it is assumed that the drug’s effect is directly related to the concentration at the effect’s site, and the drug’s plasma concentrations are in equilibrium with the free concentration at that site (steady-state). This condition applies to drugs with direct and reversible action. However, it is recommended to measure the concentration at the action site, where the drug interacts with its receptor [19]. 

To obtain the concentration at the effect site, some approaches can be used: to develop a model that incorporates variations in plasma concentrations over time, thus simulating the concentrations at that site and connecting it to the response; or maintain a constant plasma concentration through continuous intravenous infusion, and relate it to the response [22]. Currently, in addition to the use of blood plasma, other methodologies are being used to represent exposure at the site of action, such as microdialysis, use of bronchoalveolar lavage sample, use of positron emission tomography, or use of a sample of cervical or vaginal fluid, among others [23]. 

The direct response pharmacodynamic models are the fixed-effect model, linear model, log-linear model, maximum effect model (E_max_), and the maximum effect sigmoid model (sigmoid E_max_ model) [19], the last two being the most used in PK/PD modeling [24]. The sigmoid E_max_ model is shown in Equation (1):

(1) $$E=\frac{E_{\max}\times {\;C}^{n}}{{\mathrm{EC}}_{50}+C^{n}}$$

It is an expansion of the E_max_ model. Theoretically, it reflects the increase in the interactions between the drug and its receptor, when more molecules bind to the same receptor. In this equation, E represents the measured effect, $E_{\max}$ is the maximum effect possible, C is the concentration of the drug, ${\mathrm{EC}}_{50}$ is the concentration of the drug necessary to produce 50% of effect and n is the slope factor [19,21,24]. 

## 3. Use of PK/PD Models with Antimicrobials

Studies showed that a relation between pharmacokinetics and pharmacodynamic parameters allows an assessment of the potency and efficacy of the antibacterial and antifungal agents. Consequently, it will enable a great understanding of the interactions between the antimicrobials and infecting agents [15,25]. The most used approaches to develop PK/PD models with antimicrobials are minimum inhibitory concentration (MIC) and the use of the time-kill curves [24].

### 3.1. MIC-Based Approach

The minimum inhibitory concentration (MIC) is one of the most used pharmacodynamic parameters to measure the effectiveness and potency in studies with antibacterial and antifungals [15]. The definition of MIC is the lowest concentration of an antimicrobial that prevents the visual growth of a microorganism in agar or broth after 16–20 h of incubation with a standardized inoculum containing approximately 5 × 10^5^ colony-forming units (CFU) per milliliter [26]. 

Antimicrobials, in general, have three main patterns of activity. In the first scenario, the death of microorganisms is dependent on concentration. It presents extended persistent effects, and this pattern indicates that higher concentrations would kill more quickly, which, in pharmacokinetic terms, would refer to the parameters peak serum (C_max_) and area under the concentration-time curve (AUC). In the second scenario, the microorganism death is time-dependent with minimal or no persistent effects. So, higher drug concentrations would not have a better killing effect than the lower ones, implying an approach to keep them above the MIC for a sufficient time for microbial elimination. Lastly, there are time-dependent antibiotics with a prolonged post-antibiotic effect (PAE). In this case, it is essential to optimize the AUC value [16]. For some resistant bacteria, the values of mutant prevention concentration (MPC), the drug concentration required to suppress the growth of first-generation mutant bacteria, and the mutant selection window (MSW), which describes the antibiotic concentration between the MIC and the MPC, have been used to target antibiotic exposures needed to minimize the development of resistance [27].

**Figure 1:**
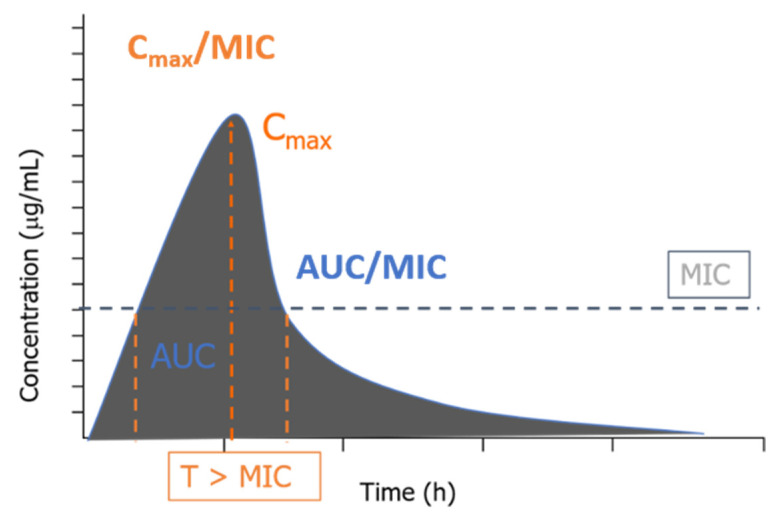
Association of the PK/PD indices with the concentration versus time profile.

When combining the pharmacodynamic (MIC) and kinetic parameters described above (C_max_ and AUC), the PK/PD indices appear (Figure 1), created to evaluate the efficacy and optimize the therapeutic regimens [15].

Despite providing a remarkable prediction about the potency of an antibiotic against a pathogen, the PK/PD indices do not give enough information about the time course of the drug’s activity. Another critical parameter is ignored when using MIC alone: persistent effects [28]. According to Mouton et al. [29], these include the PAE in vitro, which is the period of suppression of bacterial growth after exposure and subsequent artificial removal of the antimicrobial; in vivo PAE, which measures the time of bacterial growth when plasma concentrations in the serum or at the infection site fall below the MIC (sub-MIC); and the post-antibiotic sub-MIC effect (PASME), which seeks to know whether sub-MIC concentrations affect PAE in vitro (for example, prolonging this period). These effects are seen in most concentration-dependent antibiotics.

The MIC-based approach has guided antimicrobial dosage and served as a tool for doctors to make rational decisions, but this approach does have limitations. As only the free fraction of a drug (not bound to plasma proteins) has a pharmacological effect, it must reach the infection site to promote pharmacological killing. However, the MIC-based approach often disregards the effect of protein binding and tissue drug distribution, leading to therapeutic failure or antibiotic resistance [15]. Another limitation is the use of static concentrations in this modeling. When establishing the MIC value, a single one-time point is considered (between 16–20 h of incubation). It indicates only one point of concentration in which there is no visual growth. Moreover, there is interlaboratory variability in determining MIC, as it involves laboratory-dependent aspects, such as dilution factors and operator interpretation of what constitutes visual colony growth. Finally, static concentrations do not reflect in vivo conditions in which drug concentrations fluctuate between doses [15,30].

### 3.2. Use of Time-Kill Curves

A different approach to assessing the effectiveness of antimicrobials is to measure the count of viable microorganisms (MO) as a function of time and drug concentration. Over time, the microbiological killing will be observed to a greater or lesser extent depending on the concentration value and compared with the controlled growth (absence of antibiotics). The time-kill curves (TKC) are then generated, graphically plotting the amount of MO versus time (CFU/mL vs. time), thus allowing to compare the effects of different concentration profiles, in addition to providing a much more detailed PK/PD ratio than the simple use of the MIC value [31]. 

TKCs can be obtained both in vitro and in vivo. In vitro models are divided into two types: the static concentration model, in which the concentration of the antibiotic is kept constant, and the dynamic concentration model, in which various concentrations are used to simulate in vivo conditions, such as the natural decay by the drug’s half-life, fluctuations between the central and peripheral pharmacokinetic compartments, and the measurements of the free fraction at the infection site. On the other hand, the other type of model simulates concentrations obtained in vivo in a scenario of intravenous infusion with a constant flow rate [15].

The TKC models obtained in vivo are based on animal infection models already widely used in developing new agents and optimizing antibiotic therapy. The most commonly used models are thigh infection, pneumonia, pyelonephritis/kidney infection, peritonitis/septicemia, meningitis, osteomyelitis, and endocarditis. The basic premise is that the effectiveness of an antimicrobial in an animal model will be matched in humans. Nevertheless, it is known that there are essential differences in the pharmacokinetics of an antimicrobial in humans and animals, in such a way that it is necessary to simulate human PK parameters in animal models, namely the reduction of drug elimination and the control of intravenous infusion. Most models incorporate the PK/PD indices seen previously, but a PD model can be adapted that adequately models the death curves [32].

Two main types of models govern the microbial population dynamics in the modeling of TKC: the compartmental (or semi-mechanistic) model and the logistic growth model. In the first, Equation (2) describes bacterial growth and death:

(2) $$\frac{\mathrm{dN}}{\mathrm{dt}}=\left(k_{\mathrm{growth}}-k_{\mathrm{death}}\right)\times \;N$$

where N is the bacterial population, $k_{\mathrm{growth}}$ and $k_{\mathrm{death}}$ are the first-order rate constant for bacterial synthesis and the first-order rate constant for bacterial death, respectively, and dN/dt is the number of microorganisms in a culture medium as a function of time. This equation considers that the bacterial population is always homogeneous, which, in practice, is not true because, under the effect of the antibiotic, there is a tendency to select resistant strains [30].

Nolting et al. [33] used a modified model to evaluate the effects of piperacillin on *Escherichia coli* using a single-compartment in vitro dilution model with concentration profiles similar to those found in humans (use of static and dynamic concentrations). Thus, the authors adapted the experimental data using the Equation (3), which is commonly used in the evaluation of the beta-lactam pharmacodynamics [15]:

(3) $$\frac{\mathrm{dN}}{\mathrm{dt}}=\;\left(k_{o}-\frac{k_{\max\times }C^{h}}{{\mathrm{EC}}_{50}^{h}+C^{h}}\right)\times \;N$$

where EC_50_ is the potency of the drug, $k_{o}$ is the equivalent of $k_{\mathrm{growth}}$, N is the number of bacteria in CFU/mL; C is the concentration of the drug$,{\;k}_{\max}$ is the maximum kill rate constant, and h is the Hill factor previously described. It is noticed that the expression $\frac{k_{\max}\times C^{h}}{{\mathrm{EC}}_{50}^{h}+C^{}}$ is equivalent to $k_{\mathrm{death}}$, except that the latter implies natural cell death, whereas the former represents the effect of the antibiotic. To the equation, the authors also added an exponential correction factor, in the form of $\left(1-{\;e}^{-\mathrm{zxt}}\right)$, where z is a constant that considers the fact that, at the beginning of the experiment, bacterial growth has not yet been in the log phase [33] resulting in Equation (4):

(4) $$\frac{\mathrm{dN}}{\mathrm{dt}}=\left(k_{o}-\frac{k_{\max}\times {\;C}^{h}}{{\mathrm{EC}}_{50}^{h}+{\;C}^{h}}\right).\left(1-{{\;e}^{-\mathrm{zxt}}}^{}\right)\times N$$

The logistic growth model is based on the human population dynamics, which says that there is a maximum capacity in the number of individuals supported by an environment. Similarly, in an in vitro assay, the number of bacteria does not grow indefinitely, as nutrients and space for growth are limited. Thus, Equation (5) predicts this behavior, which was proposed by Mouton and collaborators [34].

(5) $$\frac{\mathrm{dN}}{\mathrm{dt}}=\left\{k_{o}\times \left(1-\frac{N}{N_{\max}}\right)\times N-k_{\max}\times \frac{{\;C}^{h}}{{\;C}^{h}\times {\mathrm{EC}}_{50}^{h}}\right\}\times N$$

where $N_{\max}$ is the maximum amount of microorganisms multiplied until reaching a plateau (stationary phase), where the multiplication speed decreases and the net growth is zero.

More complex models have emerged to simulate new conditions. In addition to the delay in bacterial growth (compensated by the exponential correction factor) and its saturation $N_{\max}$, there can be a delay in killing the three situations simultaneously. Treyaprasert and collaborators [35] used the complex model showed in Equation (6) to determine the activity of the azithromycin against strains of *Streptococcus pneumoniae, Haemophilus influenzae*, and *Moraxella catarrhalis*. The authors added the exponential correction factor $\left(1-e^{-\mathrm{yxt}}\right)$, where y describes the delay in kill:

(6) $$\frac{\mathrm{dN}}{\mathrm{dt}}=\left[k_{o}\times \left(1-\frac{N}{N_{\max}}\right)\left(1-{\;e}^{-\mathrm{zt}}\right)-\left(\frac{k_{\max}\times C^{h}}{{\mathrm{EC}}_{50}^{h}+C^{h}}\right)\left(1-{\;e}^{-\mathrm{yt}}\right)\right]\times N$$

PK/PD studies have recently been carried out, including antimicrobial resistance and a different approach when considering the plateau in the stationary phase. Some equations do not consider the emergence of resistant strains or the decrease in the speed of microbial multiplication. To overcome this, a study implemented the idea of separating bacteria into two interconnected compartments: growing population (S) and resting population (R). In the log phase, most of the bacteria would be in (S), while, as the number of bacteria reached the plateau, there would be a transformation from the stage (S) to (R); this flow is called $k_{\mathrm{SR}}$. There could also be an inverse flow so that the population dynamics, in the absence of antibiotics, were described in the Equations (7) and (8), according to Nielsen et al. [36]:

(7) $$\frac{\mathrm{dS}}{\mathrm{dt}}=k_{\mathrm{growth}}\times S-{\;k}_{\mathrm{death}}\times S-{\;k}_{\mathrm{SR}}\times S+k_{\mathrm{RS}}\times R$$

(8) $$\frac{\mathrm{dR}}{\mathrm{dt}}=k_{\mathrm{SR}}\times S-{\;k}_{\mathrm{RS}}\times R-{\;k}_{\mathrm{death}}\times R$$

where dS/dt and dR/dt are the number of bacteria in stages (S) and (R) as a function of time; $k_{\mathrm{SR}}$ and $k_{\mathrm{RS}}$ are constant transfers between the compartments; $k_{\mathrm{growth}}$ and $k_{\mathrm{death}}$ have been described previously. Then, the authors incorporated the drug’s effect, in the form of a sigmoidal model, into these two equations and evaluated the in vitro effect of constant concentrations of several antibiotics, including vancomycin, moxifloxacin, and benzylpenicillin, against *Streptococcus pyogenes*.

A further study was based on this approach and included adaptive resistance in a new PK/PD model. As is known, adaptive resistance (AR), although reversible, is a condition refractory to the bactericidal effect of an antimicrobial, being well documented in the use of aminoglycosides [37]. Thus, Mohamed et al. [38] developed a new PK/PD model and evaluated the activity of gentamicin against *Escherichia coli* through in vitro time-kill curves with static and dynamic concentrations. The authors then introduced the AR as two compartments, one active ${\mathrm{AR}}_{\mathrm{on}}$ and another dormant ${\mathrm{AR}}_{\mathrm{off}}.\;$During modeling, its presence modulated the maximum effect ($E_{\max}$ or $k_{\max}$) by the Equation (9):

(9) $$E_{\max}=E_{\max\left(0\right)}\times \left(1-\frac{{\mathrm{AR}}_{\mathrm{on}}}{{\mathrm{AR}}_{\mathrm{on}}+{\;\mathrm{AR}}_{50}}\right)$$

$E_{\max\left(0\right)}$ is the maximum effect achieved in the absence of AR, and ${\mathrm{AR}}_{50}$ describes the value of ${\mathrm{AR}}_{\mathrm{on}}$ when ${\;E}_{\max}$ is reduced by half. Finally, the authors finalized the PK/PD model (Equation (10)), incorporating the previous approach:

(10) $$\frac{\mathrm{dS}}{\mathrm{dt}}=k_{\mathrm{growth}}\times S-\left(k_{\mathrm{death}}+\frac{E_{\max}\times C^{h}}{{\mathrm{EC}}_{50}^{h}+C^{h}}\right)\times S-{\;k}_{\mathrm{SR}}\times S$$

### 3.3. In Vitro Pharmacodynamic (PD) Models 

**Figure 2:**
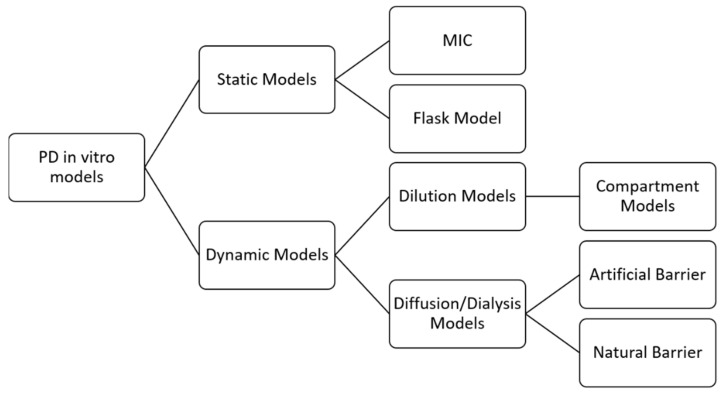
Classification of *in vitro* models. Adapted with permission from Michael et al., 2014 [39]. License Number 5352110846879, License Date 18 July 2022, Licensse Prof. Francine J Azeredo.

In vitro PD models represent one of the pillars in the conduct of PK/PD studies, and several have been developed to allow the study of death curves. The models are divided into static and dynamic models, with each type having different characteristics. Static models are easier to obtain; they present constant concentrations throughout the observed time but do not allow changes in drug or culture medium nor for measurement of post-antibiotic effects. On the other hand, the dynamic models apply changes in the concentration of the drug, which allows an extension of the observed period from 24 h to more than 72 h, if necessary. It is thus possible to measure single and multiple doses [39]. Figure 2 shows the classification of the in vitro models:

MIC is considered a parameter and a method, as it determines the lowest concentration that prevents a microorganism’s visual growth. However, as has been seen, it refers only to an outcome measure linked to a single concentration value, presenting the disadvantages already discussed. The flask model consists of some flasks with the standard bacterial/fungal inoculum is present, immersed in medium, and in which different concentrations of the antibacterial or antifungal are added depending on the corresponding MIC (e.g., 0.25 × MIC; 1 × MIC; up to high values such as 64 × MIC). The flasks are incubated at a controlled temperature and at predetermined times, samples are taken and subsequently plated, then counting the viable cells are counted as a function of time. Although these methods describe antimicrobial behavior, these models are unreliable because they do not account for clinical outcomes [15,31]. 

Unlike the two previous models, dynamic models aim to simulate in vivo conditions and may present bacterial or fungal losses (called an open-type, as there can be exchanges between the MO’s and the environment) or not (a closed-type model, in which this exchange is not possible) [40].

In dilution models, the principle of changing the concentration of the drug is to replace it with pure medium or add a medium volume to the bottle. Replacement means removing a defined volume from the in vitro model and adding an equal volume containing only the medium. In addition, the replacement can be manual, with syringe manipulation, or automatic, which is more practical as it involves peristaltic pumps. It is possible to add a filter to the system to avoid bacterial or fungal loss [40].

The dilution models can be further subdivided into models with one, two, or more compartments. One- compartment models consist of a central reservoir containing the organism, a diluent reservoir, and a disposal reservoir. The drug is administered in the central reservoir, and elimination is achieved by pumping a drug-free medium into the central reservoir; this configuration is necessary to mimic the pharmacokinetics of antibacterial and antifungal in patients (for example, the simulation of the half-life) [39].

The diffusion or dialysis model implies a closed system with no loss of bacteria or fungi. This model provides for the presence of a semipermeable membrane, permeable to the antibacterial or the antifungal, but not to the microorganisms, which separates two compartments: central (C) and peripheral (CP). Initially, the drug is in the first compartment and the microorganism is in the second. A volume containing only medium is continuously pumped into (C) so that the medium in (CP) is continuously renewed by diffusion, and changes in the drug’s concentration occur. The diffusion models can still be subdivided according to the type of membrane used. The primary examples of artificial barriers are cellulose acetate, polycarbonate, and polysulfone. For natural membranes, cell membranes and agarose gel are used [40].

## 4. Studies with PK/PD Models in Antibacterial and Antifungal

A literature review was conducted using PubMed to identify PK/PD models describing the time course of antibiotic and antifungal effects in vitro or in vivo. Inclusion criteria were limited to models based on: (i) antibacterial compounds; (ii) antifungal compounds; and (iii) time-kill curves data. Only results in the English language were considered.

A summary of all manuscripts evaluated is shown in Table 1.

### 4.1. Antibacterials

The use of PK/PD modeling contributes to the optimization of antibacterial dosage regimens, enabling a better understanding of the relationship between the drug and the microorganism, and the development of better dosage strategies for certain specific populations, as shown in the studies below:

#### 4.1.1. Beta-Lactams

##### Penicillins

The bactericidal activity of piperacillin against *Escherichia coli* was evaluated in a study with immunocompromised infected rats. The authors used a PK/PD model to assess this activity: modified sigmoidal E_max_, previously developed [33]. This model was considered adequate to evaluate the profile of bacterial reduction as a function of time to show the bactericidal effect of piperacillin. In this study, a comparison was performed between the parameters obtained through in vitro and in vivo models. With the results obtained, the importance of performing both models to assess the activity of a drug was highlighted since the in vitro model allows an understanding of the factors that can influence the pharmacological effect of the drug in the in vivo model [41].

##### Cephalosporins

In a study performed with ceftazidime, PK/PD modeling was applied to characterize bacterial death concerning ceftazidime concentrations [34]. Pharmacokinetic and pharmacodynamic data were obtained from another previously performed in vitro study that evaluated the efficacy of ceftazidime against three different strains of *Pseudomonas aeruginosa* [63].

In this study, the PK/PD model developed was a modified sigmoidal E_max_ model, which was necessary to add some factors, such as the maximum number of bacterial growth allowed by the system, adaptation, and resistance rates thus to characterize a better form the bactericidal activity of ceftazidime in the in vitro model. From the developed model, it was possible to assess the bactericidal activity of cedtazidime against *P. aeruginosa* through an appropriate description of the relationship between antibacterial concentrations and bacterial death [64].

In a study with cefaclor, the pharmacodynamics of this antibacterial were evaluated in an in vitro model, in which the bacterial species: *Escherichia coli, Moraxella catarrhalis,* and *Haemophilus influenzae* were exposed to concentrations corresponding to tissue concentrations found in humans, after oral administration. The PK and PD parameters obtained were included in the PK/PD model, which allowed the realization of different dosages for the different formulations of cefaclor: immediate-release and extended-release [44].

Cefditoren presents broad-spectrum bacterial activity against various gram-positive and gram-negative bacteria, for example, *Staphylococcus aureus* and *Streptococcus pyogenes* [65]. Matsumoto and collaborators [43] used PK/PD modeling to assess the bactericidal activity of this third-generation cephalosporin against *Streptococcus pneumoniae* and *Haemophilus influenzae*, which are considered the main etiologic agents of respiratory infections in the pediatric population. The modeling allowed the pharmacodynamic characterization of cefditoren, which mainly showed concentration-dependent and time-dependent activity against *S. pneumoniae* and *H. influenzae*, and to show time-dependent activity against a specific strain of *S. pneumoniae*. It demonstrates that antibacterials may have different activity patterns for strains of the same species [43].

##### Carbapenems

Tebipenem pivoxil, an orally administered carbapenem prodrug, was approved in Japan in 2009 [66]. In a study, PK/PD modeling of this antibacterial was performed to assess the clinical bacteriological efficacy and pharmacodynamic characteristics of *S. pneumoniae* and *H. influenzae* in the pediatric population. The PK data of this population were obtained from a population pharmacokinetic model performed in a previous study [67], and the PD data were obtained through static time-kill curves generated from an in vitro model. From the PK/PD modeling application, the study demonstrated that tebipenem pivoxil had both concentration-dependent and time-dependent activity for both bacteria. The approach was considered an adequate tool to predict bacteriological efficacy in vivo [43].

Lately, several studies have addressed the increasing resistance of microorganisms to beta-lactam antibacterials. One of the mechanisms associated with this resistance is the presence of beta-lactamase enzymes in the structure of some bacteria, which contributes to the increase of therapeutic failures in treatment with drugs of this class. It is necessary to conduct more in-depth studies on the relationship between the in vitro and in vivo susceptibility of resistant microorganisms, especially in specific populations [68]. Many authors have been developing studies with PK/PD modeling on combining a beta-lactam with another class or with a drug that can inhibit beta-lactamase, to improve its activity. In this type of study, it is crucial that during the development of the PK/PD model, parameters are added that characterize the resistance mechanism of microorganisms and the time-course of bacterial growth since using this approach, the results obtained collaborate with the better decision-making, in reduced time and offer the possibility of carrying out simulations of clinical trials evaluating the effectiveness of the combination to be used [69].

#### 4.1.2. Aminoglycosides

A study performed with gentamicin used PK/PD modeling through a modified sigmoid E_max_ model to determine a dosing regimen of gentamicin considered more suitable for patients with End-Stage Renal Disease (ESRD). The authors evaluated the activity of gentamicin against three different bacterial strains: MRSA, MSSA, and *P. aeruginosa*. The model developed allowed a more detailed description of the action of gentamicin compared to the previously defined PK/PD index and, through simulations, provided more precise information regarding the dose regimen to be indicated to patients with ESRD [45].

PK/PD modeling was performed in a study with a rat disease model of chronic lung infection, and from this model, both PK and PD parameters were obtained. In this study, the efficacy of tobramycin against *P. aeruginosa* was evaluated in different states: agar, planktonic, biofilm, and latent state. The PK/PD modeling proved to be a good and robust approach for using this model in the developing drugs for pulmonary administration. Additionally, it allowed the evaluation of different dose regimens to treat this type of infection with tobramycin [46].

#### 4.1.3. Macrolides

The bactericidal effect of azithromycin against four strains of different bacterial species was evaluated by developing a PK/PD model. Treyaprasert and collaborators [35] determined the time-kill curves of azithromycin against strains of *S. pneumoniae*, *H. influenzae*, and *Moraxella catarrhalis* using an in vitro infection model using constant concentrations for six hours. The EC_50_ values obtained from applying the models for the bacterial species *S. pneumoniae* (penicillin-intermediate), *S. pneumoniae* (penicillin-sensitive), *M. catarrhalis*, and *H. influenzae* were: 0.16, 0.05, 0.12, and 18.50 µg/mL, respectively. Although these results indicated a low activity of azithromycin against *H. influenzae*, the authors state that more studies on this antibacterial’s PK/PD relationships are needed [35].

#### 4.1.4. Rifamycins

Boutelle and collaborators [51] built a mathematical model to simulate the effect of rifampicin from the first day of treatment of tuberculosis until the last day. The results obtained from simulations with a dosage regimen of 1200 mg/day for 20 days demonstrated that the application of the PK/PD mathematical model allowed a detailed description of the antibacterial effect against *Mycobacterium tuberculosis* in the first days of treatment, in which it was possible to observe the transfer between bacterial populations from the intracellular environment to the extracellular environment, and lower efficiency of rifampicin in the intracellular environment. Furthermore, it was possible to relate the pharmacokinetic variability with the antibacterial effect of rifampicin from the model.

In another study, PK/PD modeling was used to optimize this antibacterial dose in the treatment of pulmonary tuberculosis. A combination of PK/PD modeling with multi-objective optimization was performed to solve some problems arising from rifampicin usage, such as interactions between drugs and toxicity. Combining these two methods allowed for a more detailed analysis of the simulation of dosing regimens, which may contribute to greater efficacy in the treatment, reduction of resistance, and reduction of adverse effects [49].

PK/PD modeling can also be used to determine the best PK/PD index capable of evaluating the effectiveness of an antibacterial. Lyon and Leanerts developed a PK/PD model intending to simulate the properties of rifampicin in the treating tuberculosis from data obtained from a physiologically based pharmacokinetic (PBPK) model and a model of tuberculosis infection in mice. The model obtained made it possible to mathematically describe the relationship between the drug, the bacteria, and the host. The simulations followed the results obtained in the experiments, including the determination of the PK/PD index [50].

#### 4.1.5. Oxazolidinones

A comparative analysis of the bacterial activity of linezolid against *Staphylococcus aureus* and *Enterococcus faecium* was performed. In this study, to better describe the pharmacodynamics of linezolid, the authors evaluated the effect of relative bacterial reduction. The results obtained from the analysis of the time-kill curves and the description by PK/PD modeling indicated a higher efficacy of linezolid against *S. aureus* (E_max_: 0.744 h^−1^) compared to *E. faecium* (E_max_: 0.419 h^−1^) [52].

#### 4.1.6. Fluoroquinolones

A study with moxifloxacin demonstrated that PK/PD modeling can be considered an excellent approach to determining the susceptibility breakpoint of *S. aureus* and *E. coli*. Most studies use PK/PD indices to determine susceptibility breakpoints. However, the method has several limitations, as it is based only on plasma exposure and does not consider the development of resistance. With the developed PK/PD model, it was possible to integrate clinical PK data, PD data from time-kill curves obtained from an in vitro model, and factors related to resistance development and determine the susceptibility breakpoints for the bacteria [47].

To evaluate the activity of ciprofloxacin against three different strains of *E. coli*, a PK/PD model was developed based on in vitro time-kill curves experiments, with static and dynamic concentrations. The application of the PK/PD model in this study enabled the prediction of bacterial death at different concentrations of ciprofloxacin. However, in the time-kill experiments with dynamic concentration, it was observed that the different strains grew again under certain conditions, for example, when the half-life of ciprofloxacin was 0.5 h. So, even with the limitations present in this study, the developed PK/PD model was considered an efficient approach to evaluate the resistance selection in the environment [57].

#### 4.1.7. Polymyxins

In a study carried out with patients hospitalized in the ICU, PK/PD modeling was used to predict the antibacterial activity of colistin against *P. aeruginosa*. The pharmacokinetics of colistin after aerosol administration (colistin methanesulfonate) was described in this study. With the simulations performed through the PK/PD model, it was able to predict that colistin, after aerosol administration, had a higher antibacterial activity, with an EC_50_ value equal to 25.3 µg/mL in the resistant bacterial population [53].

The use of PK/PD modeling to assess the antibacterial activity of combined dosing regimens allows for treatment optimization, contributes to increased bacterial death, and minimizes the development of resistance [70]. The bacterial effect of colistin and meropenem against *Acinetobacter baumanii* was evaluated using a semi-mechanistic PK/PD model to optimize treatment with combination therapy. This study analyzed the activity of these antibacterials in isolation and combination. It indicated that high doses of colistin would be necessary to have a more significant bactericidal effect in combined therapy. However, monitoring the plasma concentrations of colistin is necessary due to its toxicity [55]. 

In another study, the bacterial effect of this combination against *P. aeruginosa* was evaluated through an in vitro PK/PD model and simulations based on the mathematical model. With the application of the model, the authors observed the influence of the variation in the concentration of antibacterials in reducing bacterial death, especially colistin. The simulations of different dosing regimens concluded that therapy with a high dose of colistin and meropenem could be an alternative to meropenem resistance [56].

A semi-mechanistic PK/PD model was developed to evaluate the bacterial activity of polymyxin B in combination with minocycline against *Acinetobacter baumanii*. Through simulations of different dosing regimens performed using this model, the most effective dosing regimen was 1.5 mg/L minocycline + 1 mg/L polymyxin B, whose concentrations contributed to bacterial death. This combination was considered adequate to the development of resistance presented by the *A. baumanii* strain [54].

#### 4.1.8. Glycopeptides

PK/PD modeling assessed the efficacy of vancomycin. PK data from hospitalized MRSA-infected patients were correlated with PD data obtained from an in vitro study in which time-kill curves were produced at a constant concentration. The PK/PD model developed proved to be an adequate approach to characterize the relationship between the concentration of vancomycin and the bactericidal effect against MRSA and allow simulations of different dose regimens. However, this study highlighted some limitations of the in vitro model used to obtain time-kill curves, such as constant antibacterial concentration and conditions that do not entirely represent the in vivo environment [48].

#### 4.1.9. Tetracyclines

When evaluating the activity of eravacycline against *Enterobacteriaceae* isolates, the authors used an infection animal model to assess the relation between the PK/PD index (ƒAUC/MIC) and the efficacy of this antibacterial. To estimate this relation, the authors used a sigmoidal E_max_ model. The results are as follows 5.6 ± 5.0 ^h−1^ e 4.3 ± 4.0 µg/mL. These results correspond, respectively, for -1-log-kill and EC_50_, and they were considered to be acceptable, since there are similarities to the results obtained in a clinical study. The developed model demonstrates capacity of obtaining clinical efficacy [58].

### 4.2. Antifungals

PK/PD modeling is considered an essential tool for the new dosage optimization strategies in treating fungal infections, especially in situations where combined therapy is the best therapeutic option. This approach contributes to increased clinical efficacy and reduced adverse effects [71].

Studies with voriconazole used PK/PD modeling to assess its antifungal activity against some species of fungi and, based on the model obtained, simulations of different posologies. Li and collaborators [59] developed a mathematical model in which the time-kill curves data obtained for different species were fitted: *Candida albicans*, *Candida glabrata* and *Candida parapsilosis*. The time-kill curves were also simulated with pharmacokinetic parameters obtained from other studies, in which voriconazole was administered IV and orally in humans. EC_50_ values (0.02–0.05 µg/mL) were obtained from the model, which demonstrated activity with high efficacy against the species strains, and different dose regimens were simulated for IV and oral dosing regimens [59]. The model developed in this study was adapted and used to adjust the data from the dynamic time-kill curves obtained in the in vitro experiment for different strains of *C. albicans*, *C. glabrata* and *C. parapsilosis*. 

In this study, the fungal effect resulting from multiple MIC concentrations of voriconazole was evaluated, and the model demonstrated that complete inhibition occurs only at concentrations higher than 4 MIC, indicating a need to review in the clinic from which serum voriconazole concentrations are being obtained in patients with candidemia [60].

In another study carried out with voriconazole, PK/PD modeling was used to assess the intracellular antifungal activity of voriconazole against *Aspergillus fumigatus* in the prophylaxis of invasive pulmonary aspergillosis. In this modeling, extracellular voriconazole concentrations were considered in 0.0166–64 mg/L. The results showed that the cellular PK/PD model allowed the assessment of the potency of voriconazole simulated dosing regimens that can be used in prophylaxis in patients immunosuppressed patients [61].

Venisse and collaborators [62] developed PK/PD models to evaluate the fungicidal and fungistatic activity of caspofungin and fluconazole against *Candida albicans*. In the in vitro PK/PD model, the authors observed a delay in fungal growth in the presence of fluconazole. In contrast, there was a decay in the *Candida albicans* population in the presence of caspofungin. Two mechanistic models with different effect-time profiles were developed, which allowed the characterization of growth inhibition of *C. albicans* by fluconazole and death stimulation by caspofungin. Thus, PK/PD modeling proved to be an adequate approach to describe the efficiency of these two antifungals.

## 5. The Use of Pop PK/PD Models

Population pharmacokinetic/pharmacodynamic modeling (Pop PK/PD) consists of the theoretical understanding of the pharmacology of a drug and the empirical analysis of experimental data, generating a set of equations capable of describing the PK and PD of a population of individuals who take this drug [72]. The use of population PK combined with mechanism-based in vitro models are still growing in the anti-infective field. Most studies combine the PopPK with PK/PD targets to evaluate the best dosing regimen in a specific population through PTA analysis [73,74,75,76]. 

This type of modeling was applied in a study with a carbapenem drug. The pharmacokinetic profile of Meropenem in plasma and subcutaneous tissue was obtained in a study with patients with sepsis and without renal dysfunction in which a population pharmacokinetic modeling was performed [77]. This population pharmacokinetic model served as the basis for obtaining free plasma concentration-time profiles in a study that performed the PK/PD modeling of this antibacterial and aimed to evaluate the effectiveness of different meropenem dosage regimens. The PD data were obtained through an in vitro model: the hollow fiber model, in which it was possible to obtain the effect of this antibacterial in various situations of exposure to *P. aeruginosa*, and then simulations were performed for patients with augmented renal clearance, average renal clearance, and renal impairment. This approach describes the efficacy of different dosages (2, 1, and 0.5 g administered in infusions of 8 h and 30 min, with 1 g more being administered in cases of renal failure). It demonstrated the need for further studies with modeling and Monte Carlo simulations to optimize dosing regimens in patients with high renal clearance [43].

Recently, Icbal and collaborators performed this approach to evaluate tedizolid effect against *Enterococcus* spp. They simulated unbound plasma concentration-time profiles of different dosing schemes against *Enterococcus faecalis* and two clinical isolates of *Enterococcus faecium* in the hollow-fiber infection model. A PopPK approach was connected to the PKPD model and employed to predict the bacterial kinetics in plasma and target tissues over 5 days of treatment. The authors concluded that the recommended dose of 200 mg/day was insufficient to suppress bacterial growth in the system, indicating that additional factors may contribute to the clinical effect of the drug. These results corroborate to the precaution of using tedizolid in immunocompromised patients [78].

## 6. Conclusions

The application of PK/PD modeling allows studies to obtain more reliable and accurate results, which tend to be used as resources for optimizing treatments for bacterial and fungal infections. However, there is a notable difference in the number of studies with PK/PD modeling focused on antibacterials compared to antifungals. Most studies with antifungals are based only on the PK/PD indices based on the MIC. As MIC measurements only provide a static effect at a single concentration value, the results of these studies do not demonstrate the dynamic exposure of the infectious agent to the fraction of free drug at the effect’s relevant site of action. So, PK/PD modeling is the best approach to assess the effectiveness of an antibacterial or antifungal, as it establishes a relationship between drug concentration, effect, and time and consequently provides more meaningful and complete information. Therefore, more studies with PK/PD modeling are needed to assess the activity of antifungal agents and thus contribute to optimizing treatments for fungal infections. In addition, PK/PD modeling on antibacterials and antifungals can play an essential role in pharmacogenetics and model-informed precision dosing to improve patient care and diminish the toxic effect of these drugs in special populations.

## Figures and Tables

**Table 1:** Summary of time-kill curves studies using PK/PD model to evaluate antibacterial and antifungals drugs.

Author	Drug	Class	PD Model	Bacteria/Fungal	Contribution of PK/PD Modeling
DE ARAUJO et al., 2011 [41]	Piperacillin	Beta-lactam—Penicillin	In vivo	*Escherichia coli*	To model the killing effect of piperacillin against *Escherichia coli* in immunocompromised infected rats.
					To compare the PK-PD parameters obtained in vivo with those determined by simulating in vitro against *E. coli* the free tissue levels of piperacillin expected at the infection site in humans.
BERGEN et al., 2017 [42]	Meropenem	Beta-lactam—Carbapenem	In vitro	*Pseudomonas*	To quantify and characterize the relationships between meropenem concentrations, bacterial killing, and regrowth over time for a wide range of studied renal functions and do-sing regimens.
				*Aeruginosa*	
MATSUMOTO et al., 2014 [43]	Tebipenem pivoxil	Beta-lactam—Carbapenem	In vitro	*Streptococcus pneumoniae*	To predict the clinical bacteriological efficacy of antibiotics and examine the pharmacodynamics characteristics of antibiotics against bacterial strains.
				*Haemophilus influenzae*	
MOUTON; VINKS; PUNT, 1997 [34]	Ceftazidime	Beta-lactam—Cephalosporin	In vitro	*Pseudomonas aeruginosa*	To characterize in vitro bacterial killing rate as a function of ceftazidime concentrations over time.
de LA PEÑA et al., 2004[44]	Cefaclor	Beta-lactam—Cephalosporin	In vitro	*Escherichia coli*	To describe the PK/PD relationship of Cefaclor with an appropriate mathematical model and to simulate the pharmacodynamic effect of any given dose and dosing regimen on any of the bacterial strains.
				*Moraxella catarrhalis*	
				*Haemophilus influenzae*	
				*Streptococcus pneumoniae*	
MATSUMOTO et al., 2014 [43]	Cefditoren pivoxil	Beta-lactam—Cephalosporin	In vitro	*Streptococcus pneumoniae*	To predict the clinical bacteriological efficacy of cefditoren pivoxil and to examine the pharmacodynamic characteristics of antibiotics against bacterial strains.
				*Haemophilus influenzae*	
MOHAMED et al., 2011 [38]	Gentamicin	Aminoglycoside	In vitro	*Escherichia coli*	To develop a PK/PD model to describe the time course of the bactericidal activity of gentamicin against *Escherichia coli.*
ZHUANG et al., 2015 [45]	Gentamicin	Aminoglycoside	In vitro	*Staphylococcus aureus (MRSA)*	To establish the posological regimen of Gentamicin for patients with ESRD.
				*Staphylococcus aureus (MSSA)*	
				*Pseudomonas aeruginosa*	
SOU et al., 2021 [46]	Tobramycin	Aminoglycoside	In vivo	*Pseudomonas aeruginosa*	To characterize in a semi-mechanistic mathematical model in an attempt to provide a description of biofilm development and drug effects on bacteria in different states in vivo.
					To evaluate the effect of different dosing regimens with tobramycin
IQBAL et al., 2020 [47]	Moxifloxacin	Fluoroquinolone	In vitro	*Staphylococcus aureus*	To develop and evaluate a pharmacometrics approach integrating clinical PK data from (unbound) plasma and target tissues (muscle and skin) of fluoroquinolone moxifloxacin against *Staphylococcus aureus* and *Escherichia coli* in infected patients using microdialysis, as well as in vitro time-kill and resistance development.
				*Escherichia coli*	
LIM et al., 2014 [48]	Vancomycin	Glycopeptide	In vitro	MRSA	To evaluate vancomycin’s pharmacokinetics (PK) and pharmacodynamics (PD) and explore its optimal dosing regimens by modeling and simulation.
LYONS, 2014 [49]	Rifampicin	Rifamycin	In vitro	*Mycobacterium tuberculosis*	To quantitatively explore trade-offs between therapeutic and adverse effects of optimal dosing, such as rifampicin in TB-infected mice.
LYONS; LENAERTS, 2015 [50]	Rifampicin	Rifamycin	In vivo	*Mycobacterium tuberculosis*	To simulate drug therapy’s PK/PD properties for experimental TB and determine the PK/PD index that best correlates with efficacy.
GOUTELLE et al., 2011 [51]	Rifampicin	Rifamycin	In vivo	*Mycobacterium*	To set up a prototype mathematical model of TB treatment by rifampicin based on pharmacokinetics, pharmacodynamics, and disease submodels.
				*Tuberculosis*	
TREYAPRASET et al., 2007 [35]	Azithromycin	Macrolide	In vitro	*Streptococcus pneumoniae/penicillin-intermediate*	To describe the PK/PD relationship of azithromycin against different strains.
				*S. pneumoniae/penicillin-sensitive*	
				*Haemophilus influenzae*	
				*Moraxella catarrhalis*	
SCHEERANS et al., 2015 [52]	Linezolid	Oxazolidinone	In vitro	*Staphylococcus aureus*	To measure and compare the antibacterial effect of linezolid against *S. aureus* and *E. faecium* in a static in vitro infection model and characterize the underlying PK/PD relationship via a mathematical PK/PD model.
				*Enterococcus faecium*	
BOISSON et al., 2014 [53]	Colistin	Polypeptide	In vitro	*Pseudomonas aeruginosa*	To assess the effect of the route of administration on the antimicrobial effect of colistin within the lung.
ARANZANA-CLIMENT et al., 2020 [54]	Polymyxin B + Minocycline	Polypeptide + Tetracycline	In vitro	*Acinetobacter baumannii*	To develop a semi-mechanistic PK/PD model based on extensive in vitro time-kill experiments and determine the resistant bacterial count of Polymyxin B + Minocycline against *Acinetobacter baumannii.*
BIAN et al., 2019 [55]	Colistin + Meropenem	Polypeptide + Beta-lactam—Carbapenem	In vitro	*Acinetobacter baumannii*	To develop a semi-mechanistic PK/PD model to optimize the colistin and meropenem combination against carbapenem-resistant *Acinetobacter baumannii.*
MOHAMED et al., 2016 [56]	Colistin + Meropenem	Polypeptide + Beta-lactam—Carbapenem	In vitro	*Pseudomonas aeruginosa*	To develop a pharmacokinetic/pharmacodynamic (PK/PD) model that describes the in vitro bacterial time-kill curves of colistin and Meropenem alone and in combination for one wild-type and one Meropenem resistant strain of *P. aeruginosa*.
KHAN et al., 2018 [57]	Ciprofloxacin	Quinolone	In vitro	*Escherichia coli*	To predict in vitro mixed-population experiments with competition between *E. coli* wild-type and three well-defined *E. coli-*resistant mutants when exposed to ciprofloxacin.
THABIT et al., 2018 [58]	Eravacycline	Tetracycline	In vivo	*Enterobacteriaceae*	To assess the correlation of the ƒAUC/MIC index with the efficacy of eravacycline in an animal infection model and to determine its magnitude using Enterobacteriaceae.
NIELSEN et al., 2007 [36]	Benzylpenicillin Cefuroxime Erythromycin Moxifloxacin Vancomycin	Beta-lactam—Penicillin Beta-lactam— Cefalosphorin Macrolide Fluoroquinolone Glycopeptide	In vitro	*Streptococcus pyogenes*	To develop a semimechanistic PK/PD model to evaluate the antibacterial activity of different drugs against *Streptococcus pyogenes* through a time-kill curve experiment.
LI et al., 2008 [59]	Voriconazole	Azole	In vitro	*Candida albicans*	To develop a pharmacokinetic/pharmacodynamic (PK/PD) mathematical model that fits voriconazole time-kill data against *Candida* isolates in vitro and to use the model to simulate the expected kill curves for typical intravenous and oral dosing regimens.
				*Candida glabrata*	
				*Candida parapsilosis*	
LI et al., 2009 [60]	Voriconazole	Azole	In vitro	*Candida albicans*	To fit dynamic time-kill data and simulate the expected kill curves in vivo.
				*Candida glabrata*	
				*Candida parapsilosis*	
WANG et al., 2018 [61]	Voriconazole	Azole	In vitro	*Aspergillus fumigatus*	To identify a way to design an optimal prophylactic antifungal regimen through the cellular PK/PD model.
VENISSE et al., 2008 [62]	Caspofungin	Echinocandins	In vitro	*Candida albicans*	To evaluate the fungicidal and fungistatic activity of caspofungin and fluconazole against *Candida albicans.*
	Fluconazole	Azole			

PK—pharmacokinetics; PD—pharmacodynamic; MRSA—Methicillin-resistant *Staphylococcus aureus*; MSSA—Methicillin-susceptible *Staphylococcus aureus*; ESRD—End-Stage Renal Disease; TB—tuberculosis.

## Data Availability

Not applicable.
